# Candidate Genes for Yellow Leaf Color in Common Wheat (*Triticum aestivum* L.) and Major Related Metabolic Pathways according to Transcriptome Profiling

**DOI:** 10.3390/ijms19061594

**Published:** 2018-05-29

**Authors:** Huiyu Wu, Narong Shi, Xuyao An, Cong Liu, Hongfei Fu, Li Cao, Yi Feng, Daojie Sun, Lingli Zhang

**Affiliations:** 1 College of Agronomy, Northwest A&F University, Yangling 712100, China; huiyuwu@nwafu.edu.cn (H.W.); narongshi@nwafu.edu.cn (N.S.); 16631407278@163.com (X.A.); congliu@nwafu.edu.cn (C.L.); caolinwafu@126.com (L.C.); fengyi92377504@126.com (Y.F.); daojie49124098@126.com (D.S.); 2 College of Food Science and Engineering, Northwest A&F University, Yangling 712100, China; fuhongfei@nwafu.edu.cn

**Keywords:** RNA-Seq, transcription factor, chlorophyll biosynthesis precursor, carotenoid composition, wheat, yellow-green leaf color mutant

## Abstract

The photosynthetic capacity and efficiency of a crop depends on the biosynthesis of photosynthetic pigments and chloroplast development. However, little is known about the molecular mechanisms of chloroplast development and chlorophyll (Chl) biosynthesis in common wheat because of its huge and complex genome. *Ygm*, a spontaneous yellow-green leaf color mutant of winter wheat, exhibits reduced Chl contents and abnormal chloroplast development. Thus, we searched for candidate genes associated with this phenotype. Comparative transcriptome profiling was performed using leaves from the yellow leaf color type (Y) and normal green color type (G) of the *Ygm* mutant progeny. We identified 1227 differentially expressed genes (DEGs) in Y compared with G (i.e., 689 upregulated genes and 538 downregulated genes). Gene ontology and pathway enrichment analyses indicated that the DEGs were involved in Chl biosynthesis (i.e., magnesium chelatase subunit H (CHLH) and protochlorophyllide oxidoreductase (POR) genes), carotenoid biosynthesis (i.e., β-carotene hydroxylase (BCH) genes), photosynthesis, and carbon fixation in photosynthetic organisms. We also identified heat shock protein (HSP) genes (*sHSP*, *HSP70*, *HSP90*, and *DnaJ*) and heat shock transcription factor genes that might have vital roles in chloroplast development. Quantitative RT-PCR analysis of the relevant DEGs confirmed the RNA-Seq results. Moreover, measurements of seven intermediate products involved in Chl biosynthesis and five carotenoid compounds involved in carotenoid-xanthophyll biosynthesis confirmed that CHLH and BCH are vital enzymes for the unusual leaf color phenotype in Y type. These results provide insights into leaf color variation in wheat at the transcriptional level.

## 1. Introduction

Photosynthesis is the basis of plant production, and at least 90% of grain yield is determined by photosynthesis [1]. Under irradiation by light, photosynthetic pigments fix light energy and convert it into chemical energy to synthesize carbohydrates. Chlorophyll (Chl) is the primary photosynthetic pigment in higher plants, where it is responsible for light harvesting in the antenna systems and driving electron transport in the reaction centers [2]. The entire Chl biosynthetic pathway from glutamyl-tRNA to Chl *a* and Chl *b* comprises about 20 different enzymatic steps [3]. Mutations in any of these genes may lead to variations in the Chl contents [4], abnormal chloroplast development [5], and decreased photosynthetic efficiency [6], thereby yielding leaf color mutants. Mutants deficient in Chl biosynthesis have been identified in many higher plants, such as rice [7,8], *Brassica napus* [9], *Arabidopsis thaliana* [10], barley [11], and *Camellia sinensis* [12]. Many of the reported chlorotic mutants exhibit reduced Chl biosynthesis due to the lower activity of magnesium chelatase (Mg-chelatase) [11,13,14,15]. Mg-chelatase (EC 6.6.1.1) is a key regulatory enzyme that catalyzes the insertion of Mg^2+^ into protoporphyrin IX (Proto IX) in an ATP-dependent manner as the first committed step in Chl biosynthesis, where this protein complex comprises magnesium chelatase subunit I (CHLI), D (CHLD), and H (CHLH) in higher plants [16], which are all required for its activity [17]. CHLI and CHLD belong to the large family of AAA^+^ (ATPases associated with various cellular activities) proteins, but only the I-subunit has an ATPase activity [18]. The H-subunit binds the porphyrin substrate, and it is regarded as a catalytic subunit without ATPase activity [19]. The *GUN5* gene encodes the CHLH, and it has a specific function in the plastid signaling pathway where its activity is controlled by *GUN4* [20,21]. The *GUN4* gene encodes a protein that regulates Chl biosynthesis in plastids, and it has been implicated in plastid retrograde signaling via the regulation of Mg-protoporphyrin (Mg-Proto) synthesis or transport [21]. The activity of Mg-chelatase has essential regulatory roles in Chl biosynthesis and chloroplast development in higher plants. For example, in peas, the virus-induced gene silencing of *CHLI* and *CHLD* yields plants with yellow leaf phenotypes and reduced Mg-chelatase activities, as well as lower Chl accumulation correlated with undeveloped thylakoid membranes [22]. In addition, *CHLD* and *CHLI* silencing greatly reduces the levels of photosynthetic proteins, as well as being correlated with reactive oxygen species homeostasis [22]. A T-DNA insertion mutant *OsCHLH* in rice also exhibits underdeveloped chloroplasts with a low Chl content [7]. Previous studies have explored the semi-dominant leaf color mutants caused by Mg-chelatase. In barley, the mutants *chlorina-125*, *-157*, and *-161* have the same phenotypic ratio model (i.e., one green wild-type leaf, two light-green chlorina leaves, and one lethal yellow leaf at the seedling stage). The Mg-chelatase activity of the heterozygous chlorina seedlings is 25–50% of that in wild type seedlings [23,24]. In tobacco, the *Sulfur* mutant, a *CHLI* gene mutation due to the formation of inactive Mg-chelatase, is a semi-dominant aurea mutation, where homozygotes of the mutant are yellow seedling lethals, whereas the heterozygotes have reduced Chl contents and a yellow-green phenotype [25]. Moreover, a semi-dominant *CHLI* allele designated as *Oil Yellow1* (*Oy1*) has also been characterized in maize [26]. In rice, *chl1* and *chl9* mutants exhibit a yellowish-green leaf color phenotype where the abnormal leaf color is controlled by a single recessive gene. The *chl1* and *chl9* genes encode the CHLD and CHLI subunits of Mg-chelatase, and their mutation leads to underdeveloped chloroplasts and low Chl contents [13]. However, to the best of our knowledge, only a few *CHLD*, *CHLH*, and *CHLI* genes encoding the Mg-chelatase D, H, and I subunits have been reported in wheat leaf color mutants [27,28,29].

Common wheat (*Triticum aestivum* L.) is one of the most important food crops in the world. Two main types of Chl-deficient wheat mutant have been identified (i.e., albinism [30,31] and chlorina [32,33]), which have great research value for understanding the mechanisms of Chl biosynthesis and photosynthesis in wheat. However, only a few studies have reported the molecular mechanisms related to the changes in leaf color in wheat because of its large genome and high proportion of repetitive sequences (>80%) [30,34]. Most of these studies have focused on agronomic traits, photosynthetic characteristics, physiological and biochemical characteristics, and genetic mapping [31,32,33]. For example, controlled by cytogene, the wheat stage albinism mutant *FA85* exhibits albinism in cold early spring and returns to normal green gradually with the increase of temperature [35]. Chlorophyll precursors and key enzyme activities measurement of *FA85* have revealed that the accumulation of Proto IX, Mg-Proto IX, and Pchlide derives from the downregulated transcription level of Pchlide oxidoreductase and Chl synthase [36]. In addition, the five homeologous allelic chlorina mutants Driscoll’s chlorina, chlorina-1, CD3, chlorina-214, and CDd-1 exhibit the reduction in Mg-chelatase activity which leads to the accumulation of Proto IX to different extents [28]. The above five homeologous allelic chlorina mutants have been mapped on the long arm of homoeologous group 7 chromosomes [37]. In recent years, due to the development of high-throughput sequencing technology, transcriptome sequencing (RNA-Seq) has emerged as a powerful tool for studying complex biological processes at the molecular level and for identifying candidate genes involved in specific biological functions [38,39]. RNA-Seq has been employed to investigate leaf color mechanisms in various plants, such as tea (*Camellia sinensis* L.) [12], *Anthurium andraeanum* [40], *Lagerstroemia indica* [41], and wheat [34]. These studies showed that the candidate genes related to leaf color mutation were involved in chloroplast development, Chl biosynthesis, photosynthesis, and transcription factors (TFs) such as phytochrome-interacting factor (PIF1 and PIF3), Golden2-like (GLK), and v-myb avian myeloblastosis viral oncogene homolog (MYB), which might participate in the pathways identified. Therefore, RNA-Seq can increase our understanding and provide new insights into wheat leaf color mutation at the genomics level.

**Figure 1:**
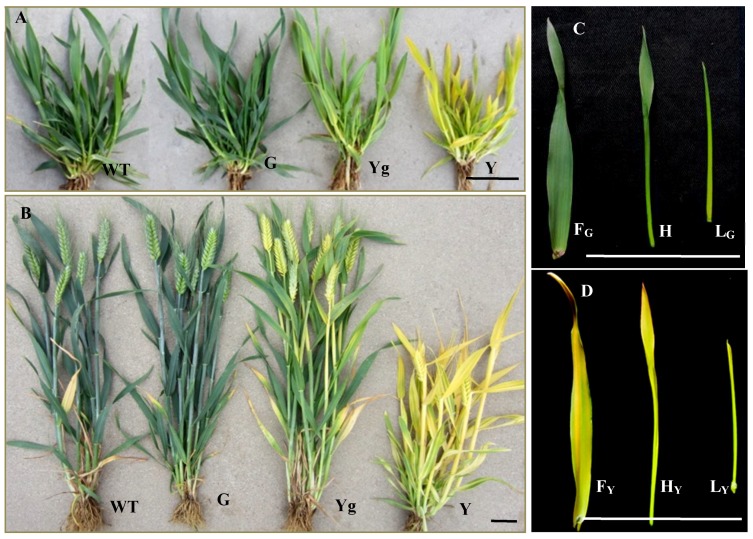
Phenotypes of the Y, Yg, and G plants among the progeny of the *Ygm* mutant and wild type (WT, Xinong1718). (**A**) Jointing stage (9 April 2016); (**B**) Adult stage (28 April 2016); (**C**) Enlarged views of the leaves in different development states in G type at the jointing stage; (**D**) Enlarged views of the leaves in different development states in Y type at the jointing stage (WT, Xinong1718). G, normal green leaf color plant in the progeny of *Ygm*; Yg, yellow-green leaf color plant in the progeny of *Ygm*; Y, yellow leaf color plant in the progeny of *Ygm*. F_G_ and F_Y_, fully-developed leaves in G and Y plants, respectively. H_G_ and H_Y_, half-developed leaves in G and Y plants, respectively. L_G_ and L_Y_, small leaf buds in G and Y plants, respectively. Bar = 5 cm.

Previously, we reported a spontaneous yellow-green leaf color mutant (*Ygm*) derived from a common wheat cultivar Xinong1718, with yellow (Y), yellow-green (Yg), and normal green (G) types under field temperature conditions from the jointing stage to the adult stage (Figure 1). Genetic analysis indicated that the yellow leaf color trait in the *Ygm* mutant was controlled by an incompletely dominant gene *Y1718* [42]. The homozygous dominant genotype (*Y1718Y1718*) of Y is extremely yellow, stunted, and sterile. The homozygous recessive genotype (*y1718y1718*) of G is normal green, whereas the heterozygous genotype (*Y1718y1718*) of Yg is yellow-green. Types Yg and G have similar agronomic traits to the wild type Xinong1718. The mutant is maintained steadily as the heterozygote genotype Yg. The *Y1718* gene was mapped on the short arm of chromosome 2B (2BS) between the simple sequence repeat marker *Xwmc25* and the expressed sequence tag-sequence tagged site marker *BE498358*, with genetic distances of 1.7 cM and 4.0 cM, respectively [42]. Thus, in the present study, the availability of a set of germplasm for the Y, Yg, and G genotypes allowed us to obtain insights into complex metabolic networks and certain biochemical traits, especially leaf color in common wheat.

In this study, comparative transcriptome profiles of the Y and G types in the progeny of the *Ygm* mutant were analyzed by RNA-Seq. Based on a combination of biochemical analysis and bioinformatics, we identified the major metabolic pathways related to leaf color and candidate genes for the loss of pigmentation in these plants. The concentrations of seven intermediate products involved in Chl biosynthesis and five carotenoid compounds involved in carotenoid-xanthophyll biosynthesis were measured to further understand the molecular mechanisms related to pigment biosynthesis in the *Ygm* mutant.

## 2. Results

### 2.1. Sequence Analysis Using RNA-Seq

To understand the molecular basis of leaf color polymorphism in the progeny of the *Ygm* mutant, cDNA libraries were constructed from the half-developed leaves of the Y and G types based on three biological replicates, which were then sequenced using the Illumina HiSeq^TM^ 2500 platform (Illumina, San Diego, CA, USA). The correlation coefficient values ranged from 0.947 to 0.989 (Table S1), thereby indicating strong correlations between the replicates. After cleaning and checking the read quality, 387,431,412 clean paired-end reads were generated, with 203,880,864 reads from the Y type and 183,550,548 from the G type, where the clean data GC content ranged from 57.29% to 59.25%, and the Q20 percentage exceeded 96.72%. The high-quality clean reads were then aligned with wheat genome sequences in the Unité de Recherche Génomique Info (URGI) database (http://wheat-urgi.versailles.inra.fr/Seq-Repository), where the alignment efficiency ranged from 69.67% to 70.71% (Table 1). Thus, the throughput and sequencing quality were sufficiently high to warrant further analysis.

### 2.2. Identification and Functional Annotation of Differentially Expressed Genes (DEGs) in G and Y

**Figure 2:**
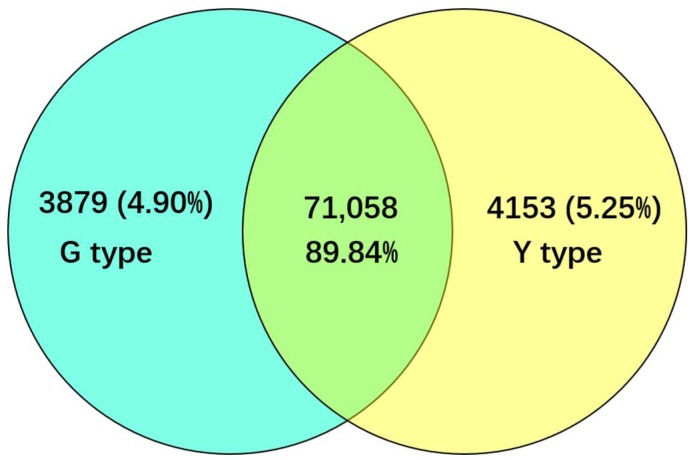
The numbers of specific genes and shared genes between G and Y.

**Figure 3:**
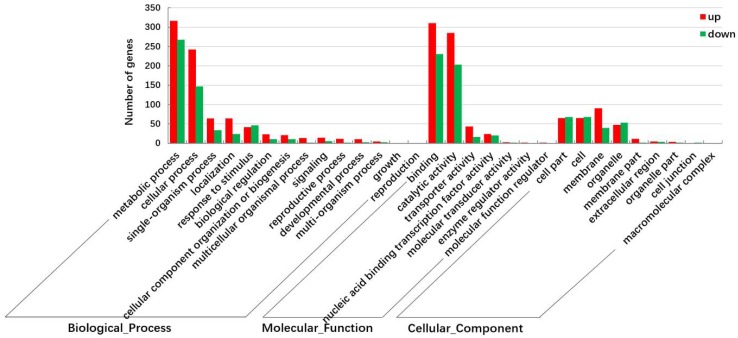
GO classifications of the DEGs in groups G and Y.

The FPKM (fragments per kilobase of transcript per million mapped reads) method was used to analyze the gene expression. As a result, 74,937 and 75,211 genes were identified respectively in the cDNA library from G and Y leaves, of which 3879 and 4153 genes were expressed specifically in the leaves of G and Y, respectively (Figure 2). In total, 1227 DEGs (false discovery rate (FDR) <0.05 and |log_2_Fold Change| (|log_2_FC|)>1) were detected, where 689 were upregulated and 538 were downregulated in Y. In order to understand the functions of the DEGs and the biological processes involved with leaf color variation, all of the DEGs were searched for in the GenBank non-redundant (Nr) protein database as well as the Gene Ontology (GO), Clusters of Orthologous Groups (COG), and Kyoto Encyclopedia of Genes and Genomes (KEGG) databases. In total, 882 DEGs had BLASTx matches with known proteins and these 882 DEGs were assigned to one or more GO terms in the biological process (685 genes), molecular function (797 genes), and cellular component (247 genes) categories. Among these three categories, the metabolic process sub-category in the biological process category accounted for the majority of the GO annotations, followed by binding and catalytic activity in the molecular function category (Figure 3). Lipid metabolic process (50 genes) and phosphorus metabolic process (20 genes), which are closely related to cell membrane functions, were significantly enriched in the biological process GO term (Table S2). The nucleic acid binding transcription factor (TF) activity and sequence-specific DNA binding TF activity were the most significantly enriched GO terms in the molecular function category (Table S2). In the cellular component category, most of the DEGs were involved with cell, cell part, and membrane components (Figure 3), but the most significantly enriched component was membrane part (Table S2).

**Figure 4:**
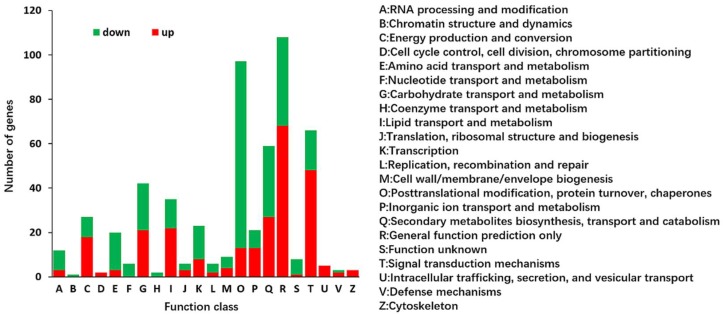
Clusters of Orthologous Groups (COG) classifications of the annotated 434 DEGs. The capital letters on the horizontal axis indicate the COG categories that are listed on the right of the histogram, and those on the vertical axis indicate the number of DEGs.

All of the 1227 DEGs were further annotated based on the COG database to obtain functional predictions and classifications (Figure 4). In total, 434 (35.37%) of the DEGs were finally mapped onto 22 different COG categories, where the “general function prediction only” cluster represented the largest group (108, 24.88%), followed by “posttranslational modification, protein turnover, chaperones” (97, 22.35%), “signal transduction mechanisms” (66, 15.20%), and “secondary metabolites biosynthesis, transport and catabolism” (59, 13.59%). In addition, “carbohydrate transport and metabolism” (42, 9.67%) and “lipid transport and metabolism” (35, 8.1%) were also annotated, which are closely related to energy metabolism and cell membrane function.

**Figure 5:**
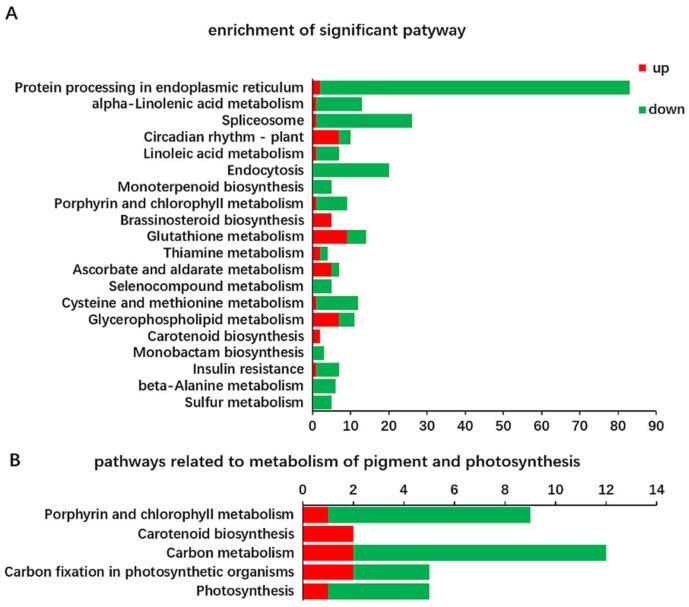
Kyoto Encyclopedia of Genes and Genomes (KEGG) classifications of DEGs. (**A**) Enrichment of the top 20 most significant pathways (*p*-value < 0.05). The vertical axis shows the annotations of the KEGG metabolic pathways. The horizontal axis represents the DEG numbers annotated in each pathway; (**B**) KEGG-based pathway assignments of the 33 DEGs (Y versus G) related to photosynthesis and pigment metabolism: photosynthesis (five genes), porphyrin and chlorophyll metabolism (nine genes), carotenoid biosynthesis (two genes), carbon fixation in photosynthetic organisms (five genes), and carbon metabolism (12 genes).

To identify biological pathways, the DEGs were annotated with the corresponding enzyme commission (EC) numbers from BLASTx alignments against the KEGG pathway databases. Among the 1227 DEGs, 323 (26.32%) were assigned to 82 KEGG pathways (Table S3), where the top 11 pathways were considered significant at a cut-off FDR corrected *p*-value (*q*-value) < 0.05 (Figure 5A). The 11 enriched pathways comprised protein processing in the endoplasmic reticulum, alpha-linolic acid metabolism, spliceosome, circadian rhythm-plant, linoleic acid metabolism, endocytosis, monoterpenoid biosynthesis, porphyrin and chlorophyll metabolism, brassinosteroid biosynthesis, glutathione metabolism, and thiamine metabolism. Most of the genes mapped in the first eight significantly enriched pathways had downregulated expression trends, except for the circadian rhythm-plant pathway. Chlorophyll and carotenoid biosynthesis are crucial for the leaf color and photosynthesis. We focused our analysis on the major genes related to pigment metabolism and photosynthesis. The results indicated that 33 genes related to either photosynthesis (five genes), Chl metabolism (nine genes), carotenoid biosynthesis (two genes), carbon fixation in photosynthetic organisms (five genes), or carbon metabolism (12 genes) pathways were differentially expressed, and the enrichment of each pathway is shown in Figure 5B. These pathways were investigated in greater detail and they may be important for the unusual leaf color phenotype in Y.

### 2.3. Identification of DEGs Related to Chl Metabolism and Carotenoid Biosynthesis in Y and G Plants

**Figure 6:**
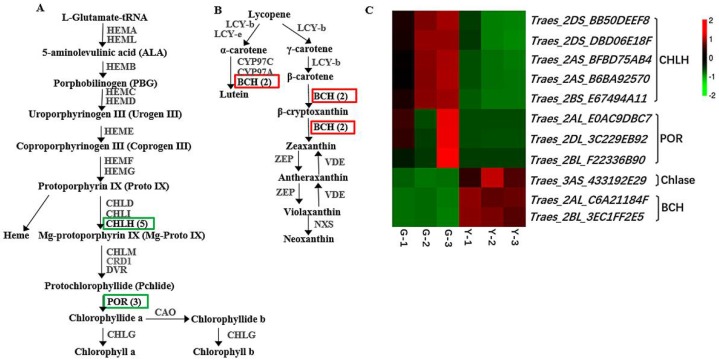
DEGs at the transcript level involved in chlorophyll and carotenoid biosynthesis pathways. (**A**) Chlorophyll biosynthesis pathway; (**B**) Carotenoid–xanthophyll biosynthesis pathway. In (**A**,**B**), upregulated genes are marked by red-line borders and downregulated genes by green-line borders. The numbers following each gene name indicate the number of corresponding DEGs identified in our database; (**C**) Expression profile clustering for chlorophyll and carotenoid biosynthesis pathways. Expression ratios are based on log_2_ FPKM values (fragments per kilobase of transcript per million mapped reads), where each vertical column represents a sample (G-1, G-2, and G-3; Y-1, Y-2, and Y-3), and each horizontal row represents a single gene. CHLH, Mg-chelatase H subunit; POR, protochlorophyllide oxidoreductase; Chlase, chlorophyllase; BCH, β-carotene hydroxylase.

To further identify the key transcripts related to yellow leaf color formation in the mutant Y type, the DEGs in two pathways for Chl metabolism and carotenoid biosynthesis were compared in detail between the Y and G transcriptomes. The results demonstrated that eight genes in the porphyrin and Chl metabolism pathway had downregulated expression levels (*q* < 0.05, fold change > 2) (Figure 6A,C), including five genes encoding CHLH (i.e., *Traes_2DS_BB50DEEF8*, *Traes_2DS_DBD06E18F*, *Traes_2AS_BFBD75AB4*, *Traes_2AS_B6BA92570*, and *Traes_2BS_E67494A11*), and three genes encoding protochlorophyllide oxidoreductase (POR), which catalyzes the conversion of proto-chlorophyllide into chlorophyllide during Chl biosynthesis (i.e., *Traes_2BL_F22336B90*, *Traes_2AL_E0AC9DBC7*, and *Traes_2DL_3C229EB92*). Only one gene (i.e., *Traes_3AS_433192E29*) encoding chlorophyllase (Chlase) was upregulated in Y. Moreover, our comparison of the DEGs involved in carotenoid biosynthesis showed that two genes encoding β-carotene hydroxylase (BCH) (substrate: β-carotene; product: zeaxanthin) in the carotenoid biosynthesis pathway were significantly upregulated in Y (Figure 6B,C). The results indicate that photosynthetic pigment biosynthesis is important for the unusual phenotype of Y.

### 2.4. Identification of DEGs Related to Photosynthesis

**Figure 7:**
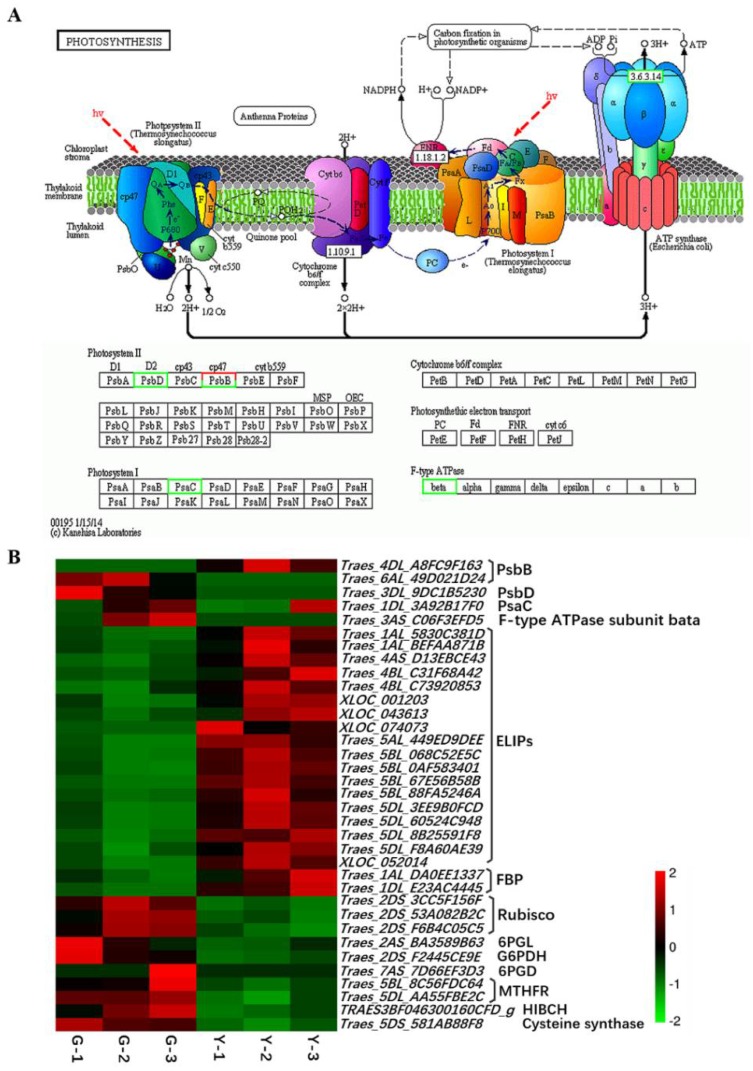
DEGs mapped onto the photosynthesis pathway. (**A**) Photosynthesis pathway. The image of the known photosynthesis pathway was obtained from the freely available KEGG database (http://www.kegg.jp/kegg-bin/show_pathway?ko00195). The green border denotes lower expression in Y compared with G, red color denotes higher expression, and half red/half green donates both up- and downregulated genes in Y compared to G. The blue dashed lines denote photosynthetic electron transport in the thylakoid membrane, red dashed lines denote light irradiation. The black dashed lines denote energy conversion of carbon fixation in photosynthetic organisms and the solid arrows denote molecular interaction or relation; (**B**) Expression profile clustering for genes involved in the photosynthesis and carbon metabolism pathway. Expression ratios are based on log_2_ FPKM values, where each vertical column represents a sample (G-1, G-2, and G-3; Y-1, Y-2, and Y-3), and each horizontal row represents a single gene. PsbB, photosystem II 47 kDa protein; PsbD, photosystem II protein D2; PsaC, photosystem I subunit VII; ELIPs, early light-inducible proteins; FBP, fructose-1,6-bisphosphatase; Rubisco, ribulose-1,5-bisphosphate carboxylase/oxygenase; 6PGL, 6-phosphogluconolactonase; G6PDH, glucose-6-phosphate 1-dehydrogenase; 6PGD, 6-phosphogluconate dehydrogenase; MTHFR, methylenetetrahydrofolate reductase; HIBCH, 3-hydroxyisobutyryl-CoA hydrolase-like protein.

The Chl content was closely related to photosynthesis. In this study, we identified five DEGs involved in photosynthesis, including two genes that encode photosystem II 47 kDa protein (PsbB), one gene that encodes photosystem II protein D2 (PsbD), one gene encode PsaC in photosystem I, and F-type ATPase β subunit, most of which were significantly downregulated in the Y type (Figure 7A,B). Moreover, most of the genes that mapped to the carbon metabolism and carbon fixation pathways in photosynthetic organisms exhibited decreased expression in Y compared with G, where the genes encoding ribulose-1,5-bisphosphate carboxylase/oxygenase (Rubisco), 6-phosphogluconolactonase (6PGL), glucose-6-phosphate 1-dehydrogenase (G6PDH), and 6-phosphogluconate dehydrogenase (6PGD) were downregulated. However, two fructose-1,6-bisphosphatase genes were upregulated (Figure 7B). In addition, 18 genes related to early light-inducible proteins (ELIPs) were significantly upregulated in Y (Figure 7B). These results indicate that changes in the expression levels of these genes might have blocked photosynthesis, thereby influencing chlorophyll biosynthesis in the mutant Y type.

### 2.5. Analysis of TFs and Heat Shock Proteins (HSPs) in Y and G Plants

**Figure 8:**
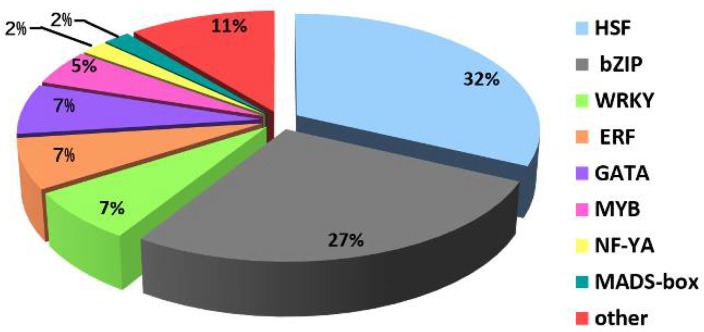
Percentages of different transcription factors involved in the “sequence-specific DNA binding transcription factor activity” GO term.

Transcription factors are important regulators that can activate or repress the expression of genes in a sequence-specific manner, thereby affecting or controlling many biological processes. We found that the GO term “sequence-specific DNA binding transcription factor activity” was most significantly enriched among the entries in the molecular function category (Table S2). Therefore, we identified all of the TFs among the DEGs in Y and G using the plant transcription factor database (PlantTFDB) v4.0 (http://planttfdb.cbi.pku.edu.cn/). In total, 44 DEGs encoding putative TFs were identified and categorized into eight different common families (Table S4). The heat shock transcription factor (HSF) protein family was the most abundant (14 TFs, 32%), where all of exhibited downregulated expression in Y (Table S4), followed by the bZIP (12 TFs, 27%), WRKY (three TFs, 7%), ERF (three TFs, 7%), and GATA (three TFs, 7%) families (Figure 8).

**Figure 9:**
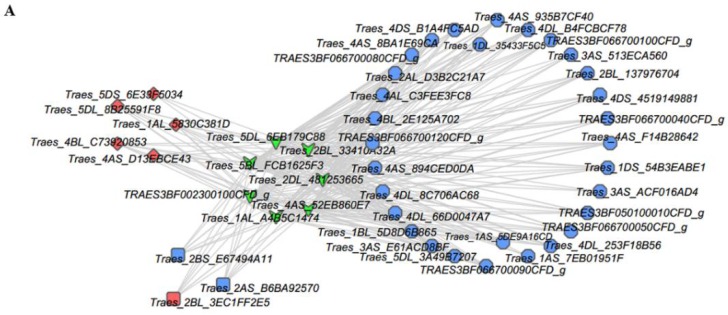

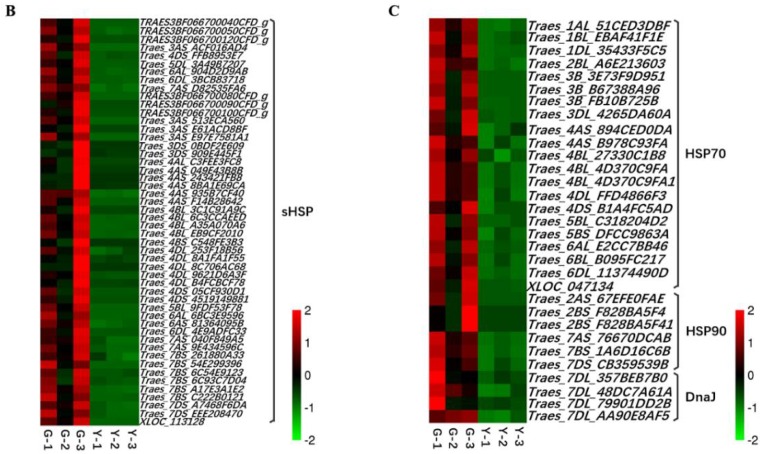
Gene interaction network diagrams and expression profile clustering for genes encoding heat shock proteins (HSPs). (**A**) Interactions between heat shock transcription factors (HSFs) and other genes. Green inverted triangles represent HSFs, HSFA6B, and HSFB2B. Pink rhombuses represent early light-inducible proteins (ELIPs). Blue circles represent HSPs. Blue squares represent CHLH. The pink square represents β-carotene hydroxylase (BCH); (**B**,**C**) Expression profile clustering for HSP encoding genes. Expression ratios are based on log_2_ FPKM values, where each vertical column represents a sample (G-1, G-2, and G-3; Y-1, Y-2, and Y-3), and each horizontal row represents a single gene. sHSP, small heat shock protein; HSP70, heat shock cognate 70 kDa protein; HSP90, heat shock 90 kDa protein; DnaJ, chaperone protein DnaJ.

In order to obtain a more intuitive understanding of the roles of those downregulated HSFs, totally, seven HSF binding motifs were projected in the TFDB database (Figure S1). The binding motifs of these seven HSFs (Figure S1) were then searched to identify the cis-acting elements present in the promoters of the DEGs using FIMO software (version 4.9.0) [43]. The results showed that many genes interacted with these seven HSF genes (Table S5). Thus, we selected 37 genes related to leaf color variations as target nodes and genes annotated as HSFs as source nodes to generate an interaction network diagram using Cytoscape software (version 3.3.0) [44]. We found that genes encoding 30 HSPs (i.e., small HSPs (sHSPs) and HSP70) comprised the main group that interacted with the seven HSFs (Figure 9A). Interestingly, most of the genes annotated as heat shock proteins (HSPs) (e.g., sHSPs, HSP70, and HSP90) in the “protein processing in endoplasmic reticulum” pathway had significantly reduced mRNA levels in the Y type (Figure 9B,C and Table S6). In addition, we found that genes encoding ELIPs, CHLH, and BCH also interacted with the seven HSFs (Figure 9A). Thus, we suggest that downregulation of these seven HSFs may affect the regulation of leaf color formation.

### 2.6. Quantitative Real-Time PCR (qRT-PCR) Analysis of Candidate Genes

**Figure 10:**
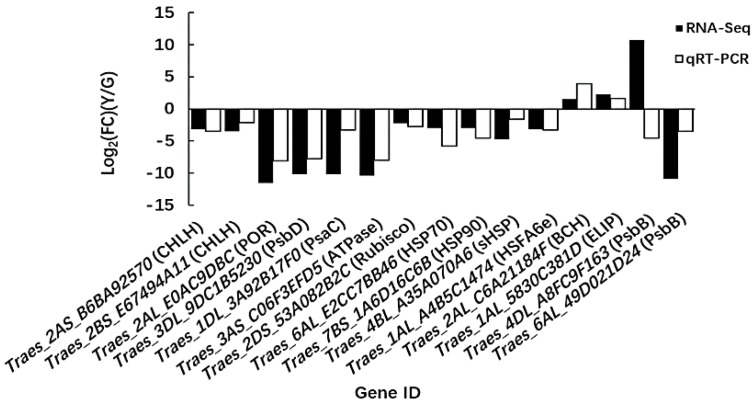
qRT-PCR validation of the RNA-Seq results for the candidate DEGs related to yellow leaf color formation in the Y type. Log_2_(FC) represents the fold change in Y relative to that in G.

To validate the reliability of the RNA-Seq data, 15 DEGs that we considered to have strong involvement with leaf color variation in Y were subjected to qRT-PCR analysis. The results showed that the expression patterns of the 14 genes detected by qRT-PCR were consistent with those in the transcriptome data, where only one gene (i.e., *Traes_4DL_A8FC9F163*) encoding PsbB had a different expression pattern (Figure 10). This discrepancy with the PsbB transcripts detected by RNA-Seq and qRT-PCR may have been caused by differences in the sensitivity of the method employed to analyze this gene. qRT-PCR is based on the specific amplification of single gene primers, and it has higher accuracy than RNA-Seq. Moreover, it is possible that this discrepancy is caused by low-expression levels of PsbB. Transcriptome results showed that *Traes_4DL_A8FC9F163* in G was almost no expression, and the expression level in Y was also low. Overall, most of the qRT-PCR data were consistent with the RNA-Seq results, thereby demonstrating the reliability of the RNA-Seq data.

### 2.7. Comparison of Chl Precursors in Y and G Plants

**Figure 11:**
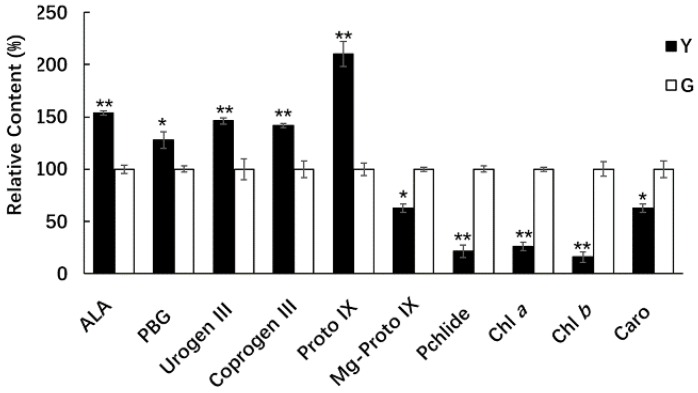
Comparison of the relative contents of chlorophyll precursors, chlorophyll, and carotenoids in H_Y_ and H_G_ leaves at the jointing stage. Three individuals were measured for each chlorophyll and chlorophyll precursor. Each plant was extracted once, and the chlorophyll contents were measured three times. Error bars indicate means ± SD based on three independent experiments. Significant differences were determined using the Student’s *t*-test in Y compared with G plants (* *p* < 0.05, ** *p* < 0.01). ALA, 5-aminolevulinic acid; PBG, porphobilinogen; Urogen III, uroporphyrinogen III; Coprogen III, coproporphyrinogen III; Proto IX, protoporphyrin IX; Mg-Proto IX, Mg-protoporphyrin IX; Pchlide, protochlorophyllide; Chl *a*, chlorophyll *a*; Chl *b*, chlorophyll *b*; Caro, carotenoid.

Mg-chelatase is encoded by the *CHLD*, *CHLI*, and *CHLH* genes in higher plants, which catalyze the insertion of Mg^2+^ into Proto IX to form Mg-Proto IX in chlorophyll biosynthesis [45] (Figure 6A). To further investigate the effects of the downregulated expression of the *CHLH* gene, which is involved in chlorophyll biosynthesis in Y plants, seven intermediate products related to the metabolic process of Chl biosynthesis were compared in half-developed leaves from the Y and G types (i.e., H_Y_ and H_G_, respectively, at the jointing stage). The 5-aminolevulinic acid (ALA), porphobilinogen (PBG), uroporphyrinogen III (Urogen III), coproporphyrinogen III (Coprogen III), and Proto IX contents of the half-developed leaves from Y plants were significantly higher than those from the G plants (Figure 11). In particular, the Proto IX contents (the substrate of Mg-chelatase) were two times higher in Y than G plants. However, the Mg-Proto IX (the product of Mg-chelatase), Pchlide, Chl *a*, and Chl *b* contents, decreased significantly in Y plants compared with G plants (Figure 11). These results indicate that the downregulated expression of the *CHLH* gene decreased the Mg-chelatase activity and reduced the production of Chl *a* and Chl *b* in Y plants.

### 2.8. Analysis of the Carotenoid Composition in Y and G Plants by HPLC

**Figure 12:**
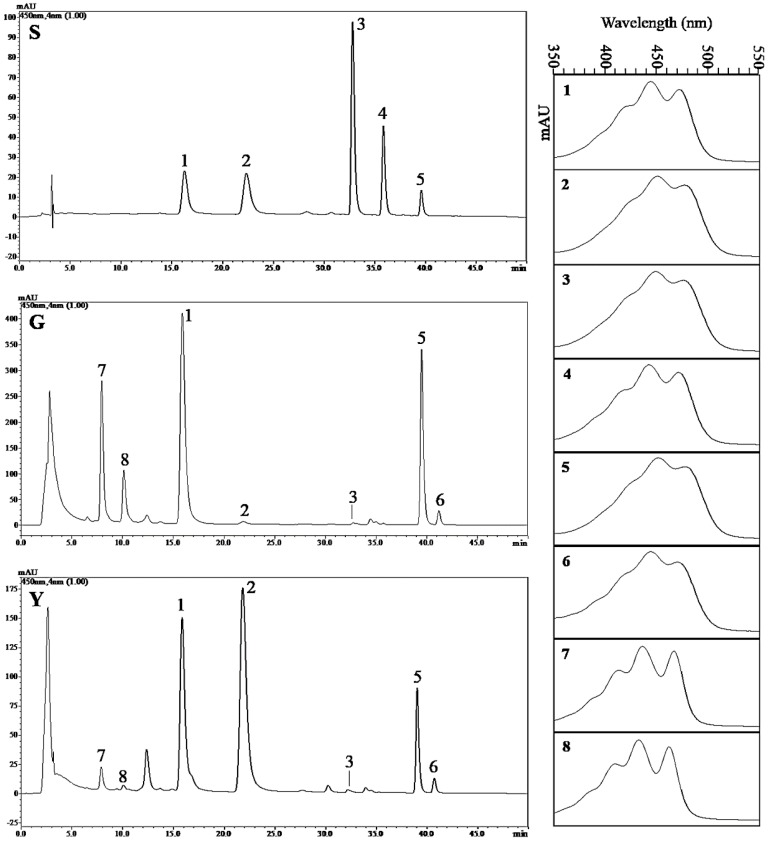
High performance liquid chromatography (HPLC) elution profiles for carotenoids accumulated in G and Y leaves at 450 nm. (**S**) HPLC elution profiles for five carotenoid standards. (**G**) HPLC elution profiles for carotenoids accumulated in G leaves. (**Y**) HPLC elution profiles for carotenoids accumulated in Y leaves. The vertical axis shows the absorbance (mAU) at 450 nm, and the horizontal axis represents the retention time for carotenoids. The right panel is the absorption spectra from peak 1 through 8 at 450 nm in Y and G types. The vertical axis shows the mAU, and the horizontal axis represents absorption wavelength. Peak 1, lutein (absorption peak λmax nm: 444, 472); peak 2, zeaxanthin (450, 476); peak 3, β-cryptoxanthin (451, 476); peak 4, α-carotene (445, 473); peak 5, β-carotene (452, 477); peak 6, 9-cis-β-carotene (446, 472); peak 7, violaxanthin (416, 438, 468); peak 8, neoxanthin (413, 435, 463).

Our results showed that the total carotenoid level was significantly reduced in Y (Figure 11), but upregulated expression of the *BCH* gene related to carotenoid biosynthesis was also identified (Figure 6B,C). To further confirm the involvement of *BCH* in carotenoid-xanthophyll biosynthesis and the yellow leaf color phenotype in Y plants, we quantitatively analyzed five carotenoid compounds (i.e., lutein, zeaxanthin, β-cryptoxanthin, α-carotene, and β-carotene) in the half-developed leaves from the Y and G types using high performance liquid chromatography (HPLC), and the results are shown in Figure 12 and Table 2. The standard curve for carotenoids is shown in Table S7. β-carotene and lutein were the major carotenoids found in G plants, and a very small amount of zeaxanthin was also detected (Figure 12). Yellow plants differed in terms of the composition and levels of carotenoids. Consistent with the increased expression of *BCH* genes, the zeaxanthin level was significantly higher (more than 40 times) in Y, and it was the most abundant carotenoid, whereas the β-carotene and lutein levels were significantly lower than those in G plants (Figure 12 and Table 2). The β-cryptoxanthin level did not differ significantly and α-carotene was below the detection limit in G and Y (Figure 12 and Table 2). In addition, violaxanthin and neoxanthin were identified in Y and G according to individual peaks in the absorption spectra, but their levels were significantly lower in Y (Figure 12). Overall, our results showed that differences in the carotenoid composition could contribute to the yellow leaf phenotype in Y.

## 3. Discussion

Leaf color formation is closely related to chloroplast development and photosynthetic pigments, and it is important for photosynthesis. Recently, many leaf color mutants have been identified in higher plants and they are valuable materials for investigating the biosynthesis of photosynthetic pigments and selective breeding for high photosynthetic efficiency. *Ygm* is a spontaneous yellow-green leaf color mutant of the cultivar Xinong1718 in common wheat, where it is an incomplete dominant semidominant mutant [42]. The dominant homozygotes (Y type) of *Ygm* have yellow leaves, whereas the heterozygotes (Yg type) have yellow-green leaves. The recessive homozygotes (G type) of *Ygm* are normal green. In this study, the G and Y types among the progeny of the *Ygm* mutant were selected as the materials, which we subjected to integrated biochemical analysis and transcriptome profiling to obtain insights into the differences in gene regulation and complex biological processes in G and Y.

### 3.1. Yellow Leaf Phenotype is Closely Associated with Chl and Carotenoid Pigment Metabolism

Leaf color variations are determined by complex biological processes. The yellow leaf color mainly depends on the Chl and carotenoid contents. Chlorophylls are essential molecules for harvesting solar energy in photosynthetic antenna systems, as well as for charge separation and electron transport within the reaction centers, where they are present in the thylakoid membrane in the form of a pigmented protein complex [2]. Comparative transcriptome profiling data for the Y type and G type among the *Ygm* mutant progeny showed that the expression of *CHLH* in Y plants was significantly downregulated compared with that in G plants (Figure 6A,C). Mg-chelatase comprises three subunits (i.e., CHLH, CHLI, and CHLD) which catalyze the insertion of Mg^2+^ into Proto IX to form Mg-Proto IX in chlorophyll biosynthesis [45]. CHLH is crucial for the Mg-chelatase activity as a catalytic subunit [19]. It has been reported that mutation of the CHLH gene leads to defective Chl and the chlorina or yellow phenotype in rice [8] and *Arabidopsis thaliana* [20]. Our analysis of seven intermediate products involved in Chl biosynthesis showed that the level of the substrate for Mg-chelatase increased significantly in Y plants whereas the level of the product from Mg-chelatase decreased significantly (Figure 11). Thus, our results indicate that the downregulated expression of the *CHLH* gene in Y plants may have decreased the Mg-chelatase activity and further reduced the production of Chl *a* and Chl *b* in Y plants.

Chl metabolism is a highly coordinated process, which is catalyzed by numerous enzymes. In addition to CHLH mentioned above, POR catalyzes the photoreduction of protochlorophyllide to chlorophyllide in Chl biosynthesis, which is a light-dependent enzyme that is present in all oxygen-producing photosynthetic organisms [46]. In our study, three genes encoding POR were significantly downregulated in Y compared with G (Figure 6A,C). Previous studies of *Arabidopsis* and rice have suggested that the Chl *a* content is directly related to the expression levels of *PORB and PORC*, and when *PORB* and *PORC* are absent in plants, the Chl *a* content is decreased and the thylakoid is not stacked [47,48]. Moreover, the *pgl10* mutant in rice has phenotypically pale-green leaves with significantly decreased Chl (Chl *a* and Chl *b*) and carotenoid contents, and less grana lamellae. Bioinformatics analysis indicates that *PGL10* encodes PORB [49]. Those results suggest that POR was inhibited in the Y type, which might have reduced the Chl content and led to the yellow leaf phenotype. Moreover, we found that one gene encoding Chlase in the Chl biosynthesis pathway was significantly upregulated in Y plants (Figure 6C). Chlase is considered to be a key enzyme in chlorophyll degradation [50,51]. However, some evidence does not support the involvement of Chlase in chlorophyll breakdown during leaf senescence [52,53,54]. For example, in *Arabidopsis*, *AtCHL1* and *AtCHL2* encode Chlase, but *CHL1* and *CHL2* single and double knockout mutants are still able to degrade chlorophyll during senescence [52]. Similarly, overexpression of the Chlase-encoding gene *ATHCOR1* in *Arabidopsis* leads to the increased breakdown of Chl *a,* but there are no substantial changes in the total amount of Chl [55]. In fact, Chlase has been shown to participate in chlorophyll breakdown in ethylene-treated citrus fruit, as well as in the tissue damage responses to fungi and bacteria [56,57,58]. The discovery of the involvement of pheophytin pheophorbide hydrolase (PPH) in early chlorophyll breakdown during leaf senescence [59] has made the biological role of Chlase controversial. Thus, the function and involvement of Chlase in Chl catabolic processes needs to be investigated further.

Carotenoids are essential components of the photosynthetic apparatus and photoprotection system, and their biosynthesis is coordinated with that of Chls in the chloroplasts [60]. In this study, HPLC analysis showed that the β-carotene and lutein levels were significantly lower in Y plants, whereas the zeaxanthin levels were more than 40 times higher than those in G plants (Figure 12 and Table 2). The changes in the abundances of carotenoids were accompanied by the altered expression of carotenoid biosynthesis genes [61]. β-carotene hydroxylase (BCH) is mainly responsible for the β-ring hydroxylation of β-carotene to produce zeaxanthin, and its activity overlaps slightly with the hydroxylation of the β-ring of α-carotene. [62]. In potato tubers, silencing the β-carotene hydroxylase genes *CHY1* and *CHY2* increases the levels of β-carotene and total carotenoids by up to 38 and 4.5 times, respectively, but reduces that of zeaxanthin [63]. By contrast, downregulating genes that encode BCH increases the β-carotene contents of sweet potato and the maize endosperm [64,65]. In our study, the expression levels of two genes that encode BCH were upregulated in Y plants (by ~1.5 and 1.8 time, respectively) (Figure 6B,C), which presumably enhanced the conversion of β-carotene into zeaxanthin, thereby increasing the zeaxanthin content and decreasing the β-carotene content. However, the increased zeaxanthin content was not converted into vioxanthin and neoxanthin, and their contents actually decreased significantly in Y plants (Figure 12). Zeaxanthin and violaxanthin are involved in the xanthophyll cycle, which is the main mechanism for photoprotection [66]. When plants are exposed to light stress, the photosynthetic organs are damaged and the conversion of violaxanthin into zeaxanthin is increased to protect against further light damage [67]. We suggest that the increase in zeaxanthin and the decrease in violaxanthin were related to damage to the photosynthetic system in Y plants, which enhanced the conversion of violaxanthin into zeaxanthin. We also found that the accumulation of lutein did not accompany the increased expression of *BCH*, and the lutein content actually decreased greatly in Y. This phenomenon has also been reported in transgenic tobacco, and it may be due to the limited effect of BCH on lutein [68]. In addition, significant reductions in the lutein content were also observed in previous studies in yellow-green tea mutants ZH1 and temperature-sensitive mutant Anji Baicha in the yellow-green stage [69,70].

### 3.2. Yellow Leaf Phenotype Affected by the Expression of Genes Related to Photosynthesis

Yellow leaf color formation is related to chloroplast development, where chloroplasts comprise the chloroplast membrane, thylakoid, and matrix, which are the main sites for photosynthesis. In higher plants, the multi-subunit pigment–protein complexes (i.e., photosystem (PS)I, PSII, light harvesting complexes, cytochrome b6/f, and ATP synthase) are embedded in the highly folded thylakoid membrane where they are responsible for light absorption and energy transfer [71,72]. The photosynthetic light reaction occurs in the thylakoid in chloroplasts, and thus we suggest that changes in gene expression might have been related to thylakoid development and photosynthesis in the two progeny of *Ygm*. Similar to the photosynthesis pathway, genes encoding PsbB (the transcriptome data indicated one upregulated gene and one downregulated gene, whereas both were downregulated according to qRT-PCR), PsbD, photosystem I subunit VII, and F-type ATPase β subunit were significantly repressed in Y plants (Figure 7). These results were consistent with our previous chloroplast ultrastructure analysis, which demonstrated that yellow leaf color formation is greatly affected by abnormal chloroplast development [42]. In addition, PSII is distributed mainly over the overlapping regions of the grana lamella [73], and the poorly stacked grana in Y might have been associated with the dramatic downregulation of the PSII protein complex [10,74,75]. In higher plants, ELIPs are light stress-induced, Chl *a*/*b*-binding proteins that accumulate in the thylakoid membranes, where their proposed function is in photoprotection [76,77,78]. The increased accumulation of ELIP transcripts and proteins is correlated with photodamage in the PSII reaction centers [79]. Overexpression of the *ELIP2* gene in *Arabidopsis* downregulates the level and activity of glutamyl tRNA reductase, CHLH, and CHLI, thereby reducing the accumulation of Chl and photosystems assembled in the thylakoid membranes [80]. In yellow leaves, we found that the accumulation of *ELIP* mRNAs was 2–4 times higher compared with that in green leaves (Figure 7B), which suggests that the yellow phenotype may be related to the upregulation of ELIP transcripts. Rubisco is a rate-limiting enzyme that participates in photosynthetic carbon fixation and it is a potential target for genetic manipulation to increase crop yields [81,82]. Albino or pale green phenotypes are observed in *Arabidopsis* transgenic lines due to the co-suppression of *Arabidopsis* Rubisco small subunit gene *RBCS3B*, where among these lines, *RBCS3B-7* exhibits abnormal thylakoid stacking and it is light sensitive under normal light [83]. Furthermore, the abundance of Rubisco is lower in *Brassica napus* and a wheat anther culture Chl-deficient mutant [30,74]. Similarly, we found that genes encoding the Rubisco small chain were significantly downregulated in Y in our study (Figure 7B). The pentose phosphate metabolic (PPP) pathway is part of the carbon metabolism pathway and some of the PPP intermediates such as ribulose-5-phosphate (Ru5P) are required for the synthesis of nucleotides, and they are shared in the carbon fixation pathways of photosynthetic organisms [84,85]. In the carbon metabolism pathway, three genes encoding glucose-6-phosphate 1-dehydrogenase (G6PDH), 6PGL, and 6PGD in the oxidative phase of PPP (product: Ru5P and DADPH) were downregulated in Y (Figure 7B). Thus, the altered expression of genes related to carbon metabolism and photosynthesis in Y may have caused abnormal chloroplast development and decreased the Chl content.

### 3.3. HSFs, HSPs, and Chloroplasts

In this study, all of the HSFs and HSPs had decreased expression levels in Y (Table S4 and Figure 9B,C). HSPs are among the most abundant protective proteins in plants where there are five conserved protein families: HSP100s, HSP90s, HSP70s, HSP60s, and sHSPs [86]. These HSPs act as molecular chaperones that participate in protein folding, assembly, and the prevention of irreversible protein aggregation to maintain cell homeostasis [87,88,89]. Therefore, HSPs play key roles in plant development and stress resistance processes. HSP-encoding genes are regulated by HSFs by specifically binding highly conserved characteristic palindromic sequence (5′-AGAAnnTTCT-3′) in the promoters of many HSP genes, thereby causing the accumulation of HSPs [90,91,92]. Seven HSFs target gene prediction analyses demonstrated that a large number of HSP genes (sHSPs and HSP70) as well as some photosynthesis and Chl biosynthesis related genes were target genes regulated by those seven HSFs (Figure 9A).

Some sHSP family members play roles in chloroplast development and photosynthesis under heat stress in many species. For example, after silencing *sHSP26* in maize, the abundances of four chloroplast proteins comprising ATP synthase subunit β, Chl *a*/*b* binding protein, oxygen-evolving enhancer protein 1, and photosystem I reaction center subunit IV declined greatly under heat stress [93]. The overexpression of *Oshsp26* in tall fescue also enhanced the photochemical efficiency of PSII (*Fv*/*Fm*) during heat stress [94]. In *Arabidopsis*, the cooperation between HSP21 and pTAC5 is required for chloroplast development under heat stress [95]. These results suggest that the inhibition of sHSPs in Y plants may be caused defective chloroplasts and led to the yellow leaf phenotype. In addition, the involvement of HSP70s with chloroplast development has been observed in *Arabidopsis* and rice. For example, suppressing the HSP70s homologues *cpHsc70-1*/*cpHsc70-2* double genes in *Arabidopsis* resulted in a white and stunted phenotype, and the chloroplasts in these plants had an unusual morphology with few or no thylakoid membranes [96]. The phenotype of the T-DNA inserted heat-sensitive rice mutant *OsHsp70CP1* varies with temperature, where a severe chlorotic phenotype and lower Chl contents are found in the leaves under a constant high temperature (40 °C), whereas plants grown at a constant temperature of 27 °C have a normal phenotype [97]. Furthermore, HSP70 has been implicated in photoprotection and the repair of PSII during and after photoinhibition [98]. DnaJ proteins are chaperones in the HSP40 family, where the J domain is generally used as a DnaJ co-chaperone to activate the HSP70 ATPase domain to allow stable substrate binding and the release of HSP70 [99,100]. In addition, DnaJ proteins are essential for normal chloroplast development. Silencing of DnaJ encoded gene *OsDjA7/8* in rice resulted in an albino lethal phenotype in the seedling stage due to disordered development of chloroplast [101]. The detection of HSPs in our transcriptome data has further proven that the expression levels of *HSPs* are closely related to chloroplast development and Chl biosynthesis in plant species.

Chloroplasts are semi-autonomous organelles and a complex network of regulatory signals exists between the nucleus and plastids. Plastid retrograde signaling is mediated by the tetrapyrrole intermediate Mg-Proto IX, and its methylester (Mg-Proto IX-ME) was identified in an *Arabidopsis* genome-uncoupled mutant (*GUN5*) that encodes the plastid-localized CHLH [20,102,103]. Mg-Proto IX can replace the action of light by inducing two nuclear HSP genes (*HSP70A* and *HSP70B*) [104]. In the present study, the Mg-Proto IX content and the expression of *HSP70* were significantly decreased in Y (Figure 9C and Figure 11), thereby indicating the possible involvement of Mg-Proto IX in the expression of HSP genes. Furthermore, in the photosynthetic system, the responses of HSPs to plastid retrograde signaling have important roles in regulating the expression of nuclear genes involved in photosynthesis. Kindgren et al. [105] found that HSP90 proteins respond to the GUN5-mediated plastid signal to control the expression of photosynthesis-associated nuclear genes (*PhANG*) during the response to oxidative stress. Recently, it was shown that *Arabidopsis* chloroplast HSP21 is activated by the GUN5-dependent retrograde signaling pathway to maintain the stability of the PSII complex and thylakoid membranes under high temperature stress by directly binding to its core subunits, such as D1 and D2 proteins [106]. These results suggest that the responses of HSPs and Mg-Proto IX to plastid signaling might have important roles in photosynthesis.

## 4. Materials and Methods

### 4.1. Plant Materials

*Ygm* was produced from a spontaneous leaf color mutant in the winter wheat cultivar Xinong1718 following 14 generations of self-pollination and direct selection for the yellow-green phenotype in each generation. The progeny of the *Ygm* mutant exhibited three leaf color phenotypes (i.e., yellow leaf plants (Y), yellow-green leaf plants (Yg), and normal green leaf plants (G)) (Figure 1). Yellow leaf plants die after the flowering stage and do not produce seeds, so this genotype was maintained by sowing seeds from the Yg plants each year. Yg plants are similar to the wild type Xinong1718 in terms of their growth period and plant height, but the yield capacity is significantly lower than that of Xinong1718. Green leaf plants are similar to the wild type Xinong1718 in terms of their growth period, plant height, and yield capacity [42]. In total, 66 plant line populations (20 and 46 derived from G and Yg plants, respectively) were sown on 2 October 2015, and 125 seeds from each plant line population were used in a single plot with a spacing of 30 cm between the rows and 8 cm between the plants, where each row measured 2 m. All of the experimental materials were grown in an experimental field at Northwest A&F University, Yangling, China, according to the standard practices employed in the local area.

The colors of the young leaves and leaf developmental states in Y and G were observed visually in the jointing stage in the field (Figure 1A). Three types of young leaves were present in the Y and G seedlings (i.e., fully-developed leaves (F_Y_ and F_G_, respectively), half-developed leaves (H_Y_ and H_G_, respectively), and small leaf buds (L_Y_ and L_G_, respectively)) (Figure 1C,D). We only collected H_Y_ and H_G_ in the jointing stage from the Y and G seedlings, respectively, to measure the concentrations of Chl precursors and carotenoids compounds, as well as for transcriptome sequencing analysis with the Illumina HiSeq™ 2500 sequencing system (Illumina, San Diego, CA, USA).

### 4.2. RNA Extraction, Library Construction, and RNA Sequencing

For transcriptome analysis, H_Y_ and H_G_ leaves were collected from Y and G seedlings at the jointing stage (31 March 2017) from 08:00 am to 10:00 am (Figure 1C,D). Six samples from three biological replicates of G and Y were used to construct cDNA libraries designated as G-1, G-2, G-3, Y-1, Y-2, and Y-3, respectively. The samples were frozen immediately in liquid nitrogen and stored at −80 °C until RNA extraction. Total RNA was extracted using Trizol Reagent (Invitrogen Life Technologies, Shanghai, China) and treated with RNase-free DNase I (TaKaRa, Dalian, China) according to the manufacturer’s instructions. The quality of the total RNA was confirmed with a NanoDrop ND1000 spectrophotometer (Thermo Scientific, Wilmington, DE, USA) coupled with 1% agarose gel electrophoresis. The RNA integrity value (>8.0) was also verified using an Agilent 2100 Bioanalyzer (Agilent Technologies, Santa Clara, CA, USA). The cDNA library construction and sequencing of six RNA samples were completed by Guangzhou GENE DENOVO Biotechnology Co., Ltd. (Guangzhou, China).

### 4.3. Sequence Alignment and Functional Annotation

In order to obtain clean data, it is essential to remove adaptor sequences, more than 10% of the unknown nucleotides, and low quality reads with more than 50% of low quality (*q*-value ≤ 20) bases. After removing rRNA using the short reads alignment tool Bowtie2 (version 2.2.9) [107], the high-quality clean reads were then mapped to the wheat reference genome sequences in the URGI database (http://wheat-urgi.versailles.inra.fr/Seq-Repository) by TopHat2 (version 2.0.3.12) [108]. In order to identify new genes and new splice variants of known genes, the transcripts were reconstructed using Cufflinks (version 2.2.1) [109] based on reference annotation based transcripts (RABT). Cufflinks constructed faux reads according to references to compensate for the influence of low coverage sequencing. During the last assembly step, all of the reassembled fragments were aligned with reference genes and similar fragments were then removed. Cuffmerge was then employed to combine the assembly results for three samples of each biological duplicate for Y and G for further downstream differential expression analysis. For functional annotation, all of the transcripts including new gene transcripts (≥200 bp and exon number >2) were annotated using the BLASTx function with protein databases, including the NCBI Nr protein database (https://ftp.ncbi.nlm.nih.gov/blast/db/FASTA/), GO (http://www.geneontology.org/), and KEGG (http://www.genome.jp/kegg/kegg2.html) databases with a significance threshold of *E* value < 10^−5^. GO annotation was conducted using Blast2GO (vision 2.5) software [110] and WEGO software (version 2.0) was then applied for gain GO function classification [111]. KEGG pathway analyses were conducted using the KEGG Automatic Annotation Server (KAAS) (http://www.genome.jp/tools/kaas/).

### 4.4. DEG Analysis

The FPKM values were used as a measure of normalized gene expression, and significance tests of differences in gene expression in Y and G (each with three biological replicates) were performed using the edgeR package (http://www.bioconductor.org/packages/release/bioc/html/edgeR.html). FDR < 0.05 and |log_2_ (fold change)| ≥ 1 were used to assess the significance of differences in gene expression. Hierarchical clustering of the DEGs was performed using the OmicShare tools, which is a free online platform for data analysis (http://www.omicshare.com/tools).

The DEGs were then annotated with the COG database to predict and classify possible functions using BLASTx (*E* value < 10^−5^). GO enrichment analysis of DEGs was implemented using the GOseq R package (Bioconductor version: release (3.7)) [112]. The enrichment of the DEGs in KEGG pathways was tested using the KOBAS software (version 2.0) [113]. GO terms and KEGG pathways with corrected *q*-values < 0.05 were considered significantly enriched for DEGs. The correlations between biological repeats in Y and G were expressed as Pearson’s correlation coefficients.

### 4.5. qRT-PCR Analysis

In order to validate the results of the RNA-Seq and DEG analyses, the color-related DEGs were selected for qRT-PCR with specific primers designed using Primer Premier 5.0 software (Table S8). cDNA was synthesized according to the manufacturer’s instructions using a PrimeScript^TM^ RT reagent kit with gDNA Eraser (TaKaRa, Dalian, China). RNA (1 μg) extracted from the half-developed leaves was used as the template. qRT-PCR was performed using a SYBR Premix Ex Taq^TM^ II Kit (TaKaRa, Dalian, China) according to the manufacturer’s instructions with a QuantStudio^®^ 7 Flex Real-Time PCR system (Applied Biosystems, Shanghai, China). Wheat 18S rRNA was used as an internal control for normalization [114]. The amplification efficiencies of the primers were checked based on standard curve analysis. Each of the reactions was performed in triplicate. Dissociation curve analysis was performed after each assay to determine the target specificity. Relative gene expression levels were calculated according to the 2^−ΔΔCt^ comparative C_T_ method [115].

### 4.6. Determination of Photosynthetic Pigments and Chl Precursors

The Chl *a*, Chl *b*, and total carotenoid contents were determined using an UV-1800 spectrophotometer (Shanghai Mapada Instruments Co. Ltd., Shanghai, China) at 645, 663, and 470 nm according to the method described by Lichtenthaler [116]. The chlorophyll biosynthesis pathway is shown in Figure 6A. Seven precursors of chlorophyll biosynthesis were examined. The ALA contents were extracted and determined as described by Dei [117]. PBG, Urogen III, and Coprogen III were quantified as described by Bogorad [118]. Proto IX, Mg-Proto IX, and Pchlide were extracted according to the methods described by Rebeiz et al. [119]. The Proto IX, Mg-Proto IX, and protochlorophyllide contents were measured with a Hitachi F-4500 fluorescence spectrometer (Hitachi Instrument (Shanghai) Co., Ltd., Shanghai, China). The wavelengths used for detecting each porphyrin were as follows: Proto IX = excitation (Ex) 400 nm and emission (Em) 633 nm; Mg-Proto IX = Ex 440 nm and Em 595 nm; and protochlorophyllide = Ex 440 nm and Em 640 nm. Three individual plants were measured from the Y and G seedlings. The H_Y_ and H_G_ leaves from each plant in the jointing stage were extracted once and each sample was measured three times. Statistical analyses were performed using Microsoft Excel 2016 (Microsoft China, Beijing, China) with the one-way ANOVA test. The concentrations of pigments and Chl precursors in the G seedlings were set to 1, and the relative values for pigments and Chl precursors in the Y samples were expressed as fold changes relative to those in the G type samples.

### 4.7. Isolation and HPLC Analysis of Carotenoid Compounds

Carotenoids were extracted from half-developed wheat leaves from Y and G plants at the jointing stage according to the method described by Norris et al. [120] with appropriate modifications. All of the extraction procedures were conducted on ice with shielding from strong light. Briefly, 2 g of the fresh leaves were ground into a powder with liquid nitrogen and saponification was performed by adding 8 mL 20% *w*/*v* KOH and methanol. The homogenates were then transferred into 50 mL centrifuge tubes and heated at 60 °C for 30 min in darkness. After cooling to room temperature, each sample was ultrasonicated with 20 mL acetone:ethyl acetate (*v*:*v* = 2:1) for 40 min at 35 °C. The extract was then centrifuged at 8000× *g* at 4 °C for 5 min. The supernatant was then transferred to a fresh tube and concentrated using a nitrogen blowing instrument. The dried extract was re-suspended in 10 mL of acetone and ethyl acetate (*v*:*v* = 2:1) and then filtered through a 0.22 μm organic membrane for HPLC analysis.

Carotenoids were separated by reverse-phase HPLC analysis on a YMC C_30_ carotenoid column (150 mm × 4.6 mm, 3 μm) (YMC Co. Ltd., Shanghai, China) using a Shimadzu LC-20A HPLC system (Shimadzu, Tokyo, Japan). HPLC separation employed (A) methanol:acetonitrile (*v*:*v* = 3:1) and (B) methyl *tert*-butyl ether with a gradient of: 0–5 min, 0% B; 5–30 min, 0–35% B; 30–40 min, 35–45% B; 40–50 min, 45–0% B. The flow rate was 1 mL/min and the column temperature was 25 °C. The detection wavelength was 450 nm. β-carotene and zeaxanthin analytical standards were purchased from Sigma–Aldrich (Shanghai, China). β-cryptoxanthin and lutein analytical standards were purchased from Extrasynthese (Lyon, France). The α-carotene analytical standard was purchased from CaroteNature (Lupsingen, Switzerland). Each standard (1 mg) was dissolved in 1 mL of dimethyl sulfoxide. In order to establish a standard curve, mixed standard solutions of 4 μg/mL, 2 μg/mL, 1 μg/mL, 0.5 μg/mL, and 0.25 μg/mL were then prepared by diluting the stock solutions. Carotenoids were identified based on their retention time relative to known standards and absorption spectra for individual peaks compared with published spectra. Individual carotenoids were quantified based on the individual peak areas by using the standard curves and expressed as μg/g by fresh weight. Three biological replicates were analyzed in Y and G plants, and the carotenoid content was expressed as the mean ± SD based on three independent determinations.

## 5. Conclusions

**Figure 13:**
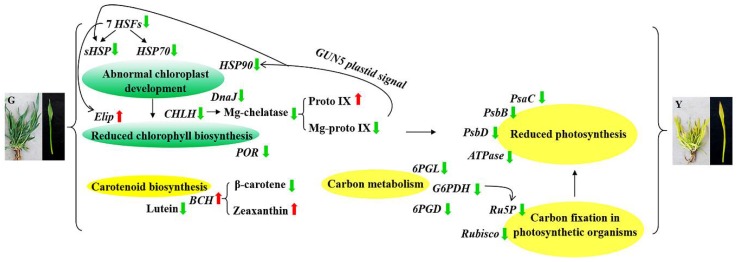
Possible formation pathway of yellow leaf phenotype of Y mutant. The red arrow indicates upregulated expression and the green arrow indicates downregulated expression. The green ovals indicate chlorophyll biosynthesis and chloroplast development. The yellow ovals indicate carotenoid biosynthesis, photosynthesis and energy metabolism.

In this study, transcriptome sequence analysis and physiological characterization were performed to identify the major molecular mechanisms related to leaf color variation in the mutant progeny of wheat *Ygm*. The transcriptome profiles differed considerably between Y and G, where various genes and pathways associated with yellow leaf formation were identified, including photosynthetic pigment synthesis, photosynthesis, and carbon fixation pathways. In addition, HSPs were shown to have important functions in response to plastid retrograde signaling and chloroplast development. Genes that interact with HSFs were shown to be associated with Chl biosynthesis. The measurements of Chl precursors indicated that the Y phenotype probably exhibited inhibited Chl biosynthesis due to the reduced activity of Mg-chelatase that is caused by the downregulation of *CHLH*. Moreover, the changes in the abundances of carotenoid composition may be associated with the yellow leaf phenotype. Overall, we speculated that the possible formation mechanism of yellow leaf phenotype in Y, which was shown in Figure 13. Our results provide new insights into the molecular mechanisms of yellow leaf formation in common wheat and they may facilitate selective breeding for high photosynthetic efficiency.

## Figures and Tables

**Table 1:** Summary of transcriptome sequencing data obtained using yellow leaves of Y plants and green leaves of G plants.

Groups	Total Reads	Clean Reads	GC (%)	Q20 (%)	Total Mapped Reads	Ratio (%)
G-1	59,480,874	58,804,014	58.37	96.76	41,285,997	70.21
G-2	61,434,418	60,676,246	58.68	96.54	42,662,324	70.31
G-3	64,966,228	64,070,288	58.68	96.65	44935,906	70.14
Y-1	67,083,278	66,278,074	59.25	96.87	46,615,892	70.33
Y-2	58,156,566	57,490,204	57.87	96.95	40,650,136	70.71
Y-3	81,071,740	80,112,586	57.29	96.61	55,817,063	69.67
Total	392,193,104	387,431,412	58.32	96.72	271,967,318	69.35

**Table 2:** Comparison of the leaf carotenoids contents (μg/g Fresh Weight) in G and Y at the jointing stage according to HPLC.

Phenotype	Lutein	Zeaxanthin	β-Cryptoxanthin	α-Carotene	β-Carotene
G	366.60 ± 23.26	3.89 ± 0.66	0.85 ± 0.24	ND	494.94 ± 45.00
Y	140.18 ± 9.43 *	157.62 ± 15.63 **	0.56 ± 0.08	ND	105.73 ± 29.75 *

The carotenoids contents are expressed as the mean ± SD based on three independent replications. Asterisks indicate significant differences in the compound measured in the Y type compared with the G type (*t*-test, *n* = 3, *: *p* < 0.05; **: *p* < 0.01). ND denotes not detected or below the detection limit for HPLC.

## Supplementary Materials

Supplementary materials can be found at http://www.mdpi.com/1422-0067/19/6/1594/s1.
