# Supplementary material for: Candidate Genes for Yellow Leaf Color in Common Wheat (Triticum aestivum L.) and Major Related Metabolic Pathways according to Transcriptome Profiling
Source: Int J Mol Sci. 2018 May 29;19(6):1594. doi: 10.3390/ijms19061594 (PMC6032196; doi:10.3390/ijms19061594)
Supplement: Supplementary file 1 [file ijms-19-01594-s001.zip › Supplementary Materials/Supplementary Fig S1.docx]

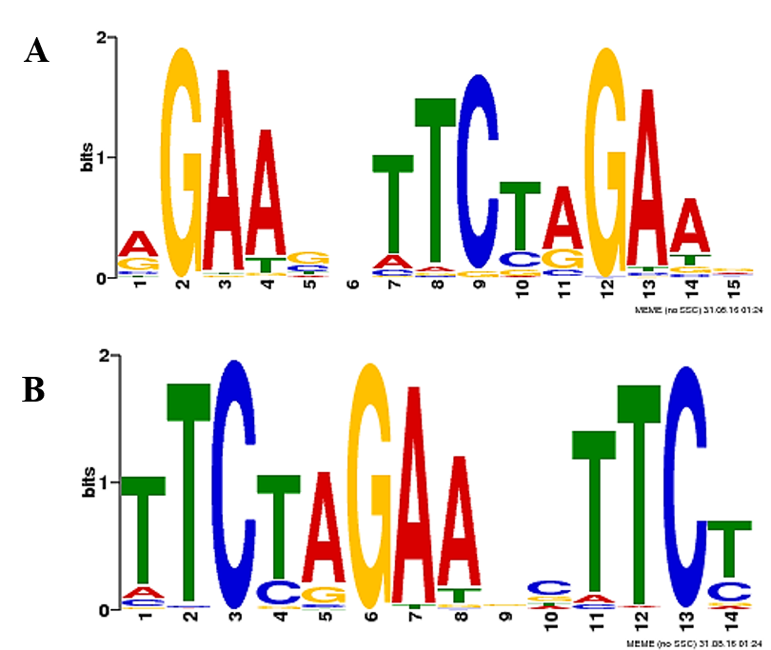


**Figure S1.** The binding motif of the HSF family protein. (A) The binding motif of *Traes_1AL_A4B5C1474*, *Traes_4AS_52EB860E7*, *TRAES3BF002300100CFD_g* and *Traes_5DL_6EB179C88* corresponding to Arabidopsis thaliana HSFA6B: (TF ID: AT3G22830.1) (B) The binding motif of *Traes_2BL_33410A32A*, *Traes_2DL_481253665* and *Traes_5BL_FCB1625F3* corresponding to Arabidopsis thaliana HSFB2B(TF ID: AT4G11660.1)
