# Supplementary material for: Candidate Genes for Yellow Leaf Color in Common Wheat (Triticum aestivum L.) and Major Related Metabolic Pathways according to Transcriptome Profiling
Source: Int J Mol Sci. 2018 May 29;19(6):1594. doi: 10.3390/ijms19061594 (PMC6032196; doi:10.3390/ijms19061594)
Supplement: Supplementary file 1 [file ijms-19-01594-s001.zip › Supplementary Materials/Supplementary Table S1.docx]

**Supplementary Table S1.** The Pearson’s correlation coefficients of biological replicates in G and Y.

| Sample | Test | Correlation | *P*-value |
| --- | --- | --- | --- |
| G | R1-vs-R2 | 0.980347 | 2.20E-16 |
|  | R1-vs-R3 | 0.977877 | 2.20E-16 |
|  | R2-vs-R3 | 0.989272 | 2.20E-16 |
| Y | R1-vs-R2 | 0.98153 | 2.20E-16 |
|  | R1-vs-R3 | 0.947346 | 2.20E-16 |
|  | R2-vs-R3 | 0.960258 | 2.20E-16 |
