# Supplementary material for: Candidate Genes for Yellow Leaf Color in Common Wheat (Triticum aestivum L.) and Major Related Metabolic Pathways according to Transcriptome Profiling
Source: Int J Mol Sci. 2018 May 29;19(6):1594. doi: 10.3390/ijms19061594 (PMC6032196; doi:10.3390/ijms19061594)
Supplement: Supplementary file 1 [file ijms-19-01594-s001.zip › Supplementary Materials/Supplementary Table S2.docx]

**Supplementary Table S2** Significantly enriched gene ontologies among downregulated or upregulated genes in Y type compared to G type (*q-value* ≤ 0.05)

| **Gene ontology (description and term)*^a^*** | **Differentially expressed genes (DEGs)** | | | |
| --- | --- | --- | --- | --- |
|  | **Total** | **Downregulated** | **Upregulated** | ***q*-value≤0.05** |
| **Cellular Component** |  |  |  |  |
| membrane part; GO:0044425 | 13 | 2 | 11 | 0.013389 |
| intrinsic component of membrane; GO:0031224 | 12 | 1 | 11 | 0.013389 |
| **Molecular function** |  |  |  |  |
| nucleic acid binding transcription factor activity; GO:0001071 | 44 | 20 | 24 | 0.000001 |
| sequence-specific DNA binding transcription factor activity; GO:0003700 | 37 | 18 | 19 | 0.000042 |
| phosphorylase activity; GO:0004645 | 3 | 1 | 2 | 0.000060 |
| catalytic activity; GO:0003824 | 488 | 203 | 285 | 0.000556 |
| purine nucleoside binding; GO:0001883 | 29 | 3 | 26 | 0.000556 |
| ribonucleoside binding; GO:0032549 | 29 | 3 | 26 | 0.000556 |
| purine ribonucleoside binding; GO:0032550 | 29 | 3 | 26 | 0.000556 |
| nucleoside binding; GO:0001882 | 29 | 3 | 26 | 0.000556 |
| carbohydrate derivative binding; GO:0097367 | 29 | 3 | 26 | 0.000591 |
| transferase activity, transferring glycosyl groups; GO:0016757 | 7 | 2 | 5 | 0.003665 |
| protein kinase activity; GO:0004672 | 13 | 1 | 12 | 0.014413 |
| phosphotransferase activity, alcohol group as acceptor; GO:0016773 | 13 | 1 | 12 | 0.018786 |
| **Biological Process** |  |  |  |  |
| response to abiotic stimulus; GO:0009628 | 35 | 11 | 24 | 0.000060 |
| Lipid metabolic process; GO:0006629 | 50 | 24 | 26 | 0.002655 |
| response to stress; GO:0006950 | 63 | 37 | 36 | 0.029433 |
| phosphate-containing compound metabolic process; GO:0006796 | 20 | 2 | 18 | 0.029433 |
| phosphorus metabolic process; GO:0006793 | 20 | 2 | 18 | 0.029433 |

*^a^*GO enrichment analysis was performed using a hypergeometric test with GOseq R package. Gene ontology categories are shown with significant FDR corrected *p*-value (*q*-value) ≤ 0.05 and the absolute value of log_2_(fold change)| ≥ 1 were used as the threshold to determine the significant DEGs.

Note: an individual gene might be assigned to more than one GO term.
