# Supplementary material for: Candidate Genes for Yellow Leaf Color in Common Wheat (Triticum aestivum L.) and Major Related Metabolic Pathways according to Transcriptome Profiling
Source: Int J Mol Sci. 2018 May 29;19(6):1594. doi: 10.3390/ijms19061594 (PMC6032196; doi:10.3390/ijms19061594)
Supplement: Supplementary file 1 [file ijms-19-01594-s001.zip › Supplementary Materials/Supplementary Table S4.docx]

**Supplementary Table S4.** Transcription factors involved in ‘sequence-specific DNA binding transcription factor activity’ GO term

| **TF family** | **Gene ID** | **Gene Annotation** | **log_2_(Fold Change)**  **Y vs. G** |
| --- | --- | --- | --- |
| HSF | *TRAES3BF002300100CFD_g* | Heat stress transcription factor A-2b | -2.4 |
|  | *Traes_1BL_5D8D6B865* | Heat stress transcription factor A-2c | -3.2 |
|  | *Traes_2AL_D3B2C21A7* | Heat stress transcription factor B-2a | -7.8 |
|  | *Traes_5DL_6EB179C88* | Heat stress transcription factor A-2e | -2.0 |
|  | *Traes_5BL_FCB1625F3* | PREDICTED: heat stress transcription factor B-2c | -1.6 |
|  | *Traes_1AL_A4B5C1474* | heat shock factor A6e, partial | -3.2 |
|  | *Traes_1DL_B5A84E4C8* | heat shock factor A6e, partial | -3.4 |
|  | *Traes_2BL_33410A32A* | heat shock factor protein 4 | -4.8 |
|  | *Traes_2DL_481253665* | heat shock factor protein 4 | -4.5 |
|  | *Traes_4BL_2E125A702* | heat shock factor A6 | -2.9 |
|  | *Traes_4BS_A05D1EF6F* | heat shock factor A6 | -2.1 |
|  | *Traes_4DL_66D0047A7* | heat shock factor HsfA2d | -2.5 |
|  | *Traes_4DL_AF19ABC7D* | heat shock factor HsfA2d | -2.0 |
|  | *Traes_4AS_52EB860E7* | heat shock factor A6 | -2.1 |
| bZIP | *Traes_2DL_5610BA574* | Protein FD | +3.1 |
|  | *Traes_5DS_6E33F5034* | Light-inducible protein CPRF2 | +2.8 |
|  | *Traes_5AS_2F996234C* | Light-inducible protein CPRF2 | +2.0 |
|  | *Traes_5DL_743B870D9* | Regulatory protein opaque-2 | +1.8 |
|  | *Traes_6BS_993CBD840* | PREDICTED: transcription factor HY5-like | +1.5 |
|  | *Traes_7DL_EECCC4DBF* | bZIP transcription factor TRAB1 | -3.0 |
|  | *Traes_7DS_C6A3C10A6* | ABSCISIC ACID-INSENSITIVE 5-like protein 6 | +2.1 |
|  | *XLOC_012690* | Protein FD | +3.7 |
|  | *XLOC_015412* | Protein FD | +2.7 |
|  | *XLOC_080553* | Protein FD | +4.0 |
|  | *XLOC_081228* | Protein FD | +3.3 |
|  | *XLOC_043307* | Protein FD | +3.0 |
| WRKY | *Traes_5BL_175E7FC38* | WRKY transcription factor 55 | -4.0 |
|  | *Traes_5DL_09F1F8F79* | WRKY transcription factor 55 | -4.0 |
|  | *TRAES3BF073300120CFD_g* | WRKY28, partial | +4.9 |
| ERF | *Traes_4AS_094442636* | root abundant factor | +5.5 |
|  | *Traes_4AS_8A64DBE8E* | ethylene-responsive element binding protein 2 | +3.3 |
|  | *Traes_6DL_FECA3FE13* | PREDICTED: ethylene-responsive transcription factor ERF054 | -3.0 |
| GATA | *Traes_6DS_77038429A* | Os02g0220400 | +2.1 |
|  | *Traes_7BL_86BB5C61E* | GATA transcription factor 24 | +2.2 |
|  | *Traes_7DL_BFADB408C* | PREDICTED: GATA transcription factor 21-like | +2.2 |
| MYB | *Traes_7DL_310E46F15* | LHY | +1.6 |
|  | *XLOC_039927* | myb-related protein, partial | +1.9 |
| NF-YA | *Traes_5BL_B5F4BA2FD* | Nuclear transcription factor Y subunit A-10 | +1.7 |
| Others | *TRAES3BF098200010CFD_g* | AP2/ERF and B3 domain-containing protein | +4.9 |
|  | *Traes_1AL_0C41C44B1* | RNA polymerase sigma factor rpoD | +1.7 |
|  | *Traes_1BL_A7A6C5DB1* | RNA polymerase sigma factor rpoD | +1.8 |
|  | *Traes_1DL_37EA23493* | RNA polymerase sigma factor rpoD | +1.9 |
|  | *Traes_7DL_D5AD8EB4B* | Multiprotein-bridging factor 1c | -3.6 |
|  | *XLOC_062860* | MADS-box transcription factor 57 | -3.0 |
