# Supplementary material for: Candidate Genes for Yellow Leaf Color in Common Wheat (Triticum aestivum L.) and Major Related Metabolic Pathways according to Transcriptome Profiling
Source: Int J Mol Sci. 2018 May 29;19(6):1594. doi: 10.3390/ijms19061594 (PMC6032196; doi:10.3390/ijms19061594)
Supplement: Supplementary file 1 [file ijms-19-01594-s001.zip › Supplementary Materials/Supplementary Table S7.docx]

**Spplementary Table S7.** The standard curve of five carotenoid standards

| **Component** | **Standard curve** | **Concentration range(μg·mL^-1^)** | **R^2^** |
| --- | --- | --- | --- |
| Lutein | y=18.282 x-0.5475 | 0.25~4 | 0.9999 |
| Zeaxanthin | y=25.912 x+0.5703 | 0. 25~4 | 0.9996 |
| β- Cryptoxanthin | y=56.011 x-0.5603 | 0.25~4 | 0.9999 |
| α-Carotene | y=31.884 x-0.4579 | 0.25~4 | 0.9998 |
| β-Crotene | y=6.5284 x+0.192 | 0.25~4 | 0.9998 |

Note: x- Concentration (μg·mL^-1^); y- Peak area (×10^-4^).
