# Supplementary material for: Candidate Genes for Yellow Leaf Color in Common Wheat (Triticum aestivum L.) and Major Related Metabolic Pathways according to Transcriptome Profiling
Source: Int J Mol Sci. 2018 May 29;19(6):1594. doi: 10.3390/ijms19061594 (PMC6032196; doi:10.3390/ijms19061594)
Supplement: Supplementary file 1 [file ijms-19-01594-s001.zip › Supplementary Materials/Supplementary Table S8.docx]

**Spplementary Table S8.** Primer pair sequences used in qRT-PCR

| **Gene ID** | **sequence (5’ – 3’)** | **Description** |
| --- | --- | --- |
| *Traes_2AS_B6BA92570-F* | TTTGATGCAGGGAAGTCGCA | Magnesium-chelatase subunit H(CHLH) |
| *Traes_2AS_B6BA92570-R* | CTTGTCTGGTGGGAAGCTGA |  |
| *Traes_2BS_E67494A11-F* | TTCGACTCAGACCCGACGAA | Mg-chelatase subunit XANTHA-F |
| *Traes_2BS_E67494A11-R* | CACCTGTGCATTTGCTGTGG |  |
| *Traes_2AL_E0AC9DBC7-F* | GCTTCCTCACCAACTCGCT | Protochlorophyilide reductase(POR) |
| *Traes_2AL_E0AC9DBC7-R* | GAGCTCTGACGGACGACTG |  |
| *Traes_2AL_C6A21184F-F* | TGCCGCTCACAAGATACACC | β-carotene hydroxylase A1(BCH) |
| *Traes_2AL_C6A21184F-R* | GTCCTCCAGCTCCTTGGGTC |  |
| *Traes_6AL_49D021D24-F* | CTGTAAATCCAGCGTGGGGT | Photosystem II 47 kDa protein(PsbB) |
| *Traes_6AL_49D021D24-R* | CCCAATGTACCCGCAGCAAT |  |
| *Traes_4DL_A8FC9F163-F* | TCGTGGGGTGGTTGGAGTA | Photosystem II 47 kDa protein(PsbB) |
| *Traes_4DL_A8FC9F163-R* | CTGCCAAGAAACACAAGCCA |  |
| *Traes_3DL_9DC1B5230-F* | ACTGGTTACGAAGGGACCGT | Photosystem II protein D2(PsbD) |
| *Traes_3DL_9DC1B5230-R* | AACCAACCCCCTAAAGCGAA |  |
| *Traes_3AS_C06F3EFD5-F* | AGACGATTTGACCGACCCTG | ATP synthase CF1 beta subunit (plastid) |
| *Traes_3AS_C06F3EFD5-R* | CCAACGATCCGAGGCTGTAA |  |
| *Traes_1DL_3A92B17F0-F* | AGATGTGAATCTGCCTGCCC | NADH dehydrogenase subunit(PsaC) |
| *Traes_1DL_3A92B17F0-R* | AGAGCCATGCTACGTGTTGT |  |
| *Traes_2DS_53A082B2C-F* | GTTCGAGACCCTGTCTTACTTG | Ribulose bisphosphate carboxylase small chain PW9, chloroplastic(Rubisco) |
| *Traes_2DS_53A082B2C-R* | AAGCCAACCTTGCTGAACT |  |
| *Traes_1AL_5830C381D-F* | CAAGTCGAGCGGAATCTGGA | High molecular mass early light-inducible protein HV58(ELIP) |
| *Traes_1AL_5830C381D-R* | AGATGTTGACGAAGGGAGCG |  |
| *Traes_6AL_E2CC7BB46-F* | TCTACTCCACCATCACCCGT | Heat shock cognate 70 kDa protein 1(HSP70) |
| *Traes_6AL_E2CC7BB46-R* | GTCCATCTTTGCGTCCCTCA |  |
| *Traes_7BS_1A6D16C6B-F* | GAGTTCATCAGCTATCCCATCTC | Heat shock protein 90(HSP90) |
| *Traes_7BS_1A6D16C6B-R* | CCCTCCTCAGTATCCTTCTTCT |  |
| *Traes_4BL_A35A070A6-F* | GCATCGACTGGAAGGAGAC | PREDICTED: 17.9 kDa class I heat shock protein-like(sHSP) |
| *Traes_4BL_A35A070A6-R* | CGCTGATCTGGAGGATGTT |  |
| *Traes_1AL_A4B5C1474-F* | GAGGTTGGTGAGTTTGGATTTG | Heat shock factor A6e, partial(HSFA6e) |
| *Traes_1AL_A4B5C1474-R* | CTCCTGCCTTAGCTTCACTAC |  |
| *18srRNA-F* | GTGACGGGTGACGGAGAATT | 18srRNA |
| *18srRNA-R* | GACACTAATGCGCCCGGTAT |  |
